# Conserved and divergent patterns of expression of DAZL, VASA and OCT4 in the germ cells of the human fetal ovary and testis

**DOI:** 10.1186/1471-213X-7-136

**Published:** 2007-12-18

**Authors:** Richard A Anderson, Norma Fulton, Gillian Cowan, Shona Coutts, Philippa TK Saunders

**Affiliations:** 1 Division of Reproductive and Developmental Sciences, Queen's Medical Research Institute, Edinburgh EH16 4TJ, UK; 2 MRC Human Reproductive Sciences Unit, Queen's Medical Research Institute, Edinburgh EH16 4TJ, UK

## Abstract

**Background:**

Germ cells arise from a small group of cells that express markers of pluripotency including OCT4. In humans formation of gonadal compartments (cords in testis, nests in ovary) takes place during the 1st trimester (6–8 weeks gestation). In the 2nd trimester germ cells can enter meiotic prophase in females whereas in males this does not occur until puberty. We have used qRTPCR, Westerns and immunohistochemical profiling to determine which of the germ cell subtypes in the human fetal gonads express OCT4, DAZL and VASA, as these have been shown to play an essential role in germ cell maturation in mice.

**Results:**

OCT4 mRNA and protein were detected in extracts from both 1st and 2nd trimester ovaries and testes. In ovarian extracts a marked increase in expression of VASA and DAZL mRNA and protein occurred in the 2nd trimester. In testicular extracts VASA mRNA and protein were low/undetectable in 1st trimester and increased in the 2nd trimester whereas the total amount of DAZL did not seem to change. During the 1st trimester, germ cells were OCT4 positive but did not express VASA. These results are in contrast to the situation in mice where expression of Vasa is initiated in Oct4 positive primordial germ cells as they enter the gonadal ridge. In the 2nd trimester germ cells with intense cytoplasmic staining for VASA were present in both sexes; these cells were OCT4 negative. DAZL expression overlapped with both OCT4 and VASA and changed from the nuclear to the cytoplasmic compartment as cells became OCT4-negative. In males, OCT4-positive and VASA-positive subpopulations of germ cells coexisted within the same seminiferous cords but in the ovary there was a distinct spatial distribution of cells with OCT4 expressed by smaller, peripherally located, germ cells whereas DAZL and VASA were immunolocalised to larger (more mature) centrally located cells.

**Conclusion:**

*OCT4*, *DAZL *and *VASA *are expressed by human fetal germ cells but their patterns of expression are temporally and spatially distinct. In the 1st trimester OCT4 was detected in most germ cells. In the 2nd trimester the onset of expression of *VASA *was associated with the formation of oocytes and spermatogonia both of which were OCT-4 negative. Relocation of DAZL from nucleus to cytoplasm paralleled the down regulation of OCT4 and the onset of expression of VASA. These data reveal similarities between the expression of key regulatory proteins within germ cells as they mature in male and female fetal human gonads suggesting that in the female these maturational changes are not determined by entry into meiosis.

## Background

The ovaries and testes both develop from an identical structure, the embryonic genital ridge [1]. Germ cells do not originate from within the genital ridges but differentiate from clusters of cells located in the extra-embryonic proximal epiblast [2]. Primordial germ cells (PGC) proliferate and migrate via the embryonal endoderm reaching the gonadal ridges at about 5^th ^week of pregnancy in the human (reviewed in [3]). Within the genital ridge germ cells have been shown to express the pluripotency markers OCT4 (POU5F1, [4]) and NANOG [5] and human embryonic germ cell lines, capable of differentiating into a number of cell lineages, have been established from early gonadal tissue [6,7]. We have previously reported that during the 1^st ^trimester of pregnancy the germ cells (gonocytes) in the testicular cords all express OCT4 but that only a subpopulation of germ cells remains OCT4 positive during the 2nd trimester [8]. In previous studies OCT4 positive germ cells were detected in the cortical region of human fetal ovaries recovered during the 2nd trimester [9]; however expression of OCT4 has not been investigated in the 1st trimester ovary.

Studies in knockout mice have identified a number of genes the expression of which is critical for germ cell survival and functional maturation in both ovary and testis. One such gene is *Dazl*, an RNA binding protein that is a member of a conserved gene family members of which include *BOULE *and *DAZ *[10,11]. A second example is *Mvh *(mouse Vasa homologue) a gene that encodes an RNA helicase which is specific to the germ cell lineage [12]. In mice *Dazl *mRNA is first detectable in post-migratory germ cells on e11.5 [11,13] and the protein has been immunolocalised to the cytoplasm of fetal and adult germ cells [14]. Targeted deletion of the *Dazl *gene results in germ cell loss in both males and females [14]. In *Dazl *-/- females germ cell loss occurred in fetal ovaries at the time of meiotic entry and adult ovaries did not contain oocytes [14,15]. In *Dazl *-/- males the pattern of germ cell loss is variable and in some studies has been reported to occur during fetal life [11] whilst in others it was associated with spermatogonial differentiation [14,16] or entry to meiosis [17].

*VASA *is a member of the DEAD box family of genes first identified in *Drosophila *where it was shown to be essential for female germ cell development [18]. In mice with targeted deletions of *Mvh *ovarian function appeared normal but males were infertile with demise of germ cells at the zygotene stage of meiosis [19]. Mvh has been detected in germ cells on e12.5 immediately after colonisation of the gonad but not in migratory PGCs [12,19,20]. Information on expression of VASA in human fetal gonads is limited but protein was detected in OCT4 negative cells in a testis at 21 weeks gestation [21]. First trimester ovaries have not been examined but in the 2nd trimester VASA has been localised to oocytes within primordial follicles [9].

In the present paper we have extended our studies on the germ cell subpopulations within the human fetal ovaries and testes to determine when and where DAZL and VASA proteins are expressed and have performed co-staining for OCT4 as this is commonly used to delineate the germ 'stem cell' population [8,22,23]. These studies have revealed a dynamic and overlapping pattern in the expression of these germ cell markers, revealed that nuclear localisation of DAZL parallels the onset of VASA expression, and highlighted similarities in the development of male and female germ cell lineages.

## Results

### Expression of mRNAs for *OCT4*, *DAZL *and *VASA *in first and second trimester gonads

**Figure 1:**
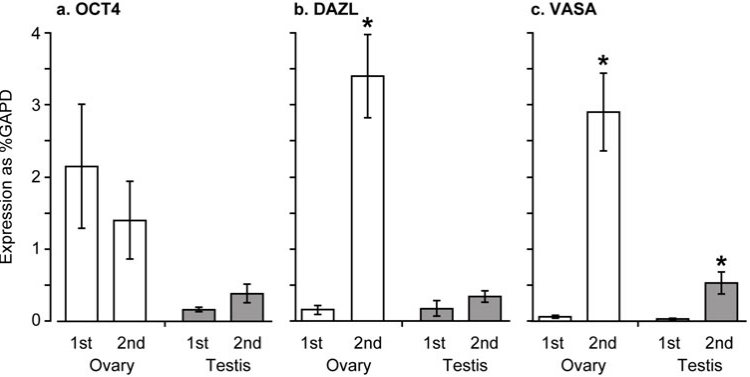
Expression of mRNAs for *OCT4*, *DAZL *and *VASA *in 1st and 2nd trimester ovary and testis. Open columns, 1st trimester, shaded columns 2nd trimester. Concentrations of mRNA are all relative to that of *GAPD *in the same samples. * P < 0.001 vs 1st trimester. Mean ± sem, n = 5–18 per group.

In ovaries *OCT4 *mRNA was detected in both the 1st and 2nd trimester samples with a slight, but non-significant decrease in the older samples (Figure 1A). In contrast the amount of mRNA for *DAZL *and *VASA *was very low in 1st trimester ovaries but was markedly increased in the 2nd trimester *DAZL *20 fold and *VASA *50 fold (both P < 0.001, Figure 1B,C),. Analysis of individual ovarian samples showed a very high correlation between expression of *DAZL *and *VASA *mRNAs (Spearman correlation coefficient 0.74, P < 0.0001). In the testis, *OCT4 *and *DAZL *mRNAs were detectable in both 1st and 2nd trimester with no significant difference between ages (Figure 1A, B). Expression of *VASA *mRNA in the testis was low/barely detectable in the 1st trimester and showed a significant increase in the 2nd trimester (p = 0.001) although this was less marked than that seen in ovarian samples (Figure 1C).

### Western analysis of total protein levels

**Figure 2:**
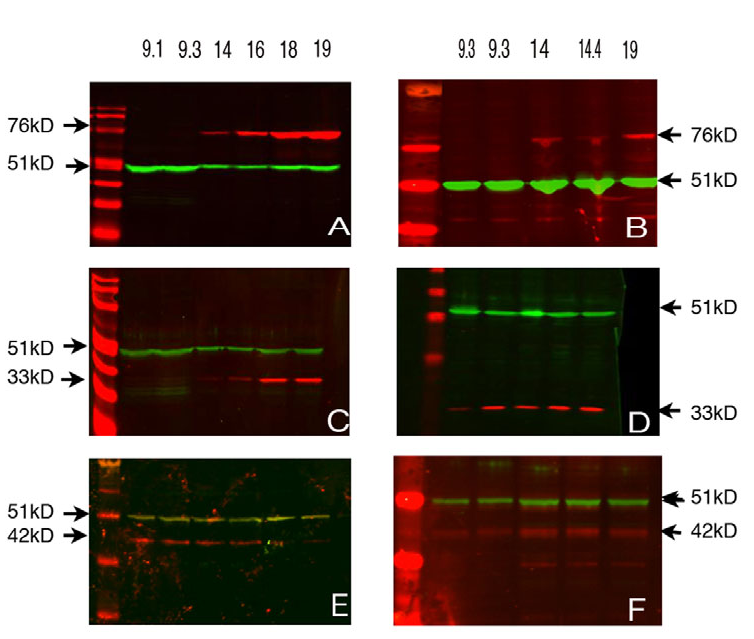
**Western analysis of 1st and 2nd trimester ovaries and testes**. In both ovarian (A) and testicular (B) samples VASA (76 Kd) was not detectable in the 1st trimester samples but was present in those from the 2nd trimester. DAZL (33 Kd) was low/undetectable in 1st trimester ovaries (C) whereas it was detectable in ovarian samples from 2nd trimester and testicular samples from both 1st and 2nd trimester (D). OCT4 (42 Kd) was present in both ovaries (E) and testes (F) during both the 1st and 2nd trimester. The loading control in all cases was β-tubulin (51 Kd).

In both ovaries and testes VASA protein was not detected in samples from the 1st trimester (Figure 2A, B) but an increase in the amount of VASA was noted during the 2^nd ^trimester (14–19 weeks) which was particularly striking in the ovarian samples (Figure 1A, B).

DAZL protein was detected in both 1st and 2nd trimester testes (Figure 2D) but only in the 2nd trimester ovaries (Figure 2C). OCT4 was detected in extracts from ovaries and testes obtained during both 1st and 2nd trimesters (Figure 2E, F).

### Immunolocalisation of OCT4, DAZL and VASA revealed differential patterns of expression in both ovary and testis

**Figure 3:**
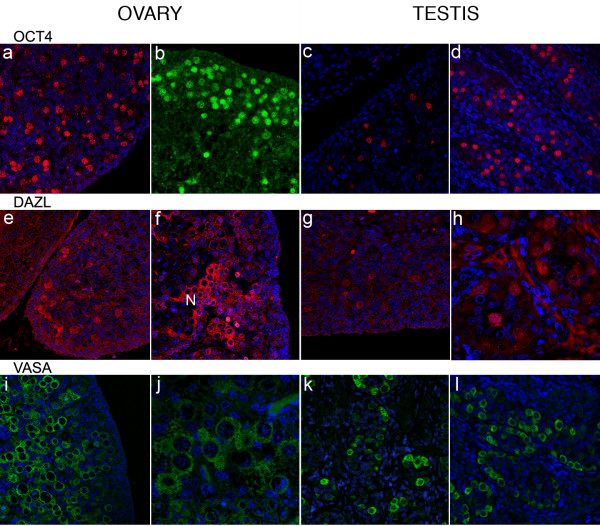
**Immunoexpression of OCT4, DAZL and VASA**. OCT4 positive germ cell nuclei were detectable in both the 1st and 2nd trimester ovaries (a, 62 d; b, 16 wk) and testes (c, 64 d; d, 16 wk). DAZL positive germ cells were rare in the 1st trimester (e, ovary 61 d; g, testis 64 d) but groups of cells ('nests', labelled N) with cytoplasmic staining were present in the 2nd trimester ovaries (f, 20 wk). During the 2nd trimester VASA protein was detected in the cytoplasm of female germ cells (i, 14 wk; j, 18 wk) throughout the ovary with the exception of the sub-epithelial layer. In the testes (k, 15 wk; l, 16 wk) VASA-positive germ cells were found in all cords.

Immunoexpression of OCT4, DAZL and VASA was germ cell specific in all samples. OCT4 positive germ nuclei were detected in both 1st and 2nd trimester ovaries (Figure 3a, b) and testes (Figure 3c, d). In both sexes immunopositive cells were distributed throughout the organ during the 1st trimester. In 2nd trimester testes OCT4 positive germ cells were detected in all seminiferous cords whereas in the ovary expression of OCT4 positive nuclei was largely confined to the peripheral cortex (Figure 3b). DAZL was immunolocalised to the nuclei of germ cells in 1st trimester gonads in both sexes. In 2nd trimester ovaries DAZL immunostaining was largely cytoplasmic and appeared to be most prominent in groups of cells (N in Figure 3f), whereas in the testes germ cells with nuclear or cytoplasmic staining were detected throughout the organ (Figure 3h). No VASA immunopositive cells were detected in 1st trimester gonads from either sex (not shown). In the 2nd trimester ovaries VASA staining was cytoplasmic and more intense in germ cells less peripherally located than those expressing OCT4 (Figure 3i, j); in testes VASA positive cells were distributed throughout the organ.

#### Co-localisation of OCT4 and DAZL

**Figure 4:**
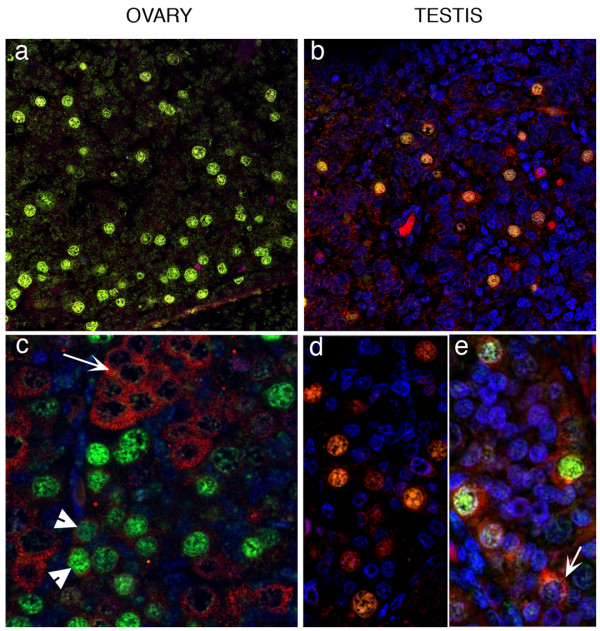
**Co-localisation of OCT4 and DAZL**. In 1st trimester ovaries (a, 61 d) and testes (b, 64 d) OCT4 (green) and DAZL (red) were co-localised to germ cell nuclei. In ovaries from the 2nd trimester (c, 14 wk) DAZL protein was almost exclusively cytoplasmic and was largely localised to OCT4 negative groups of cells (arrow); a few OCT4 positive cells had a low level of DAZL immunoexpression in their cytoplasm (arrowheads). Testes, panel b, 64 d; panel d, 16 wk; panel e, 19 wk gestation. In 2nd trimester testes (d, 16 wk; e, 19 wk) DAZL was still expressed in the nuclei of some OCT4 positive germ cells but this pattern of expression was variable with DAZL protein present in the cytoplasm of OCT4 positive and OCT4 negative (arrow panel e) cells.

In 1st trimester ovaries and testes (Figure 4a and 4b, respectively) OCT4 and DAZL were co-expressed in germ cell nuclei. In the 2nd trimester ovaries (Figure 4c) germ cells containing intense positive nuclear immunoreactivity for OCT4 were immunonegative for DAZL; in a few cells with low levels of OCT4 (Figure 4c arrowheads) a small amount of cytoplasmic DAZL was detected. In contrast, intense immunoexpression of DAZL was detected in the cytoplasm of less peripherally located groups of oogonia (arrow). However in 2nd trimester testes DAZL immunoexpression was heterogeneous with nuclear (Figure 4d) as well as cytoplasmic staining in OCT4 positive cells as well as cytoplasmic expression in cells that were OCT4 negative (Figure 4e, arrow).

#### Differential expression of OCT4 and VASA

**Figure 5:**
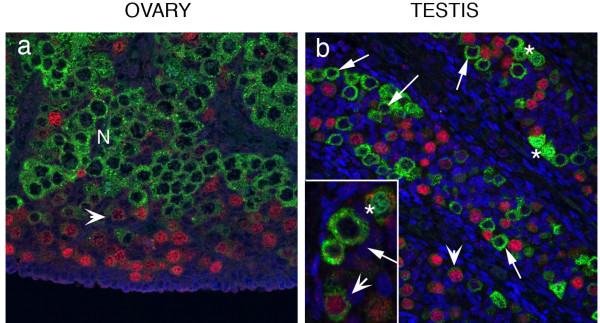
**Co-localisation of OCT4 and VASA in 16 week ovary and testes**. Panel a, ovary, cells with intense immunopositive staining for OCT4 are found at the periphery of the organ (red nuclei), VASA was detected in the cytoplasm and was most intense in cells located in nests (N) closer to the centre of the ovary. An intermediate population of cells with low intensity nuclear staining for OCT4 and low intensity staining for VASA (arrowheads) was also present. Panel b, testis, OCT4 positive and VASA positive germ cells were found within the same seminiferous cords; germ cells with intense nuclear OCT4 expression (red nuclei) were VASA negative and those with intense cytoplasmic expression of VASA (e.g. arrowed in inset panel b) were OCT 4 negative. Two other populations of male germ cells were identified, a population with low intensity immunoexpression of OCT4 which also had low intensity staining for VASA (arrowheads) and cells with nuclear VASA expression (asterisks) which were typically found in pairs.

In 1st trimester ovaries and testes all germ cells contained OCT4 positive nuclei but were immunonegative for VASA (not shown). In the 2nd trimester, OCT4 positive germ cells were located at the periphery of the ovary whereas the most intense immunoexpression of VASA was detected in the cytoplasm of OCT4 negative germ cells (oogonia) located in nests (Figure 5a, labelled N) in the central region. Expression of OCT4 and VASA was almost mutually exclusive although some cells in a transition zone between the peripheral cells that contained intense OCT4 staining and the nests of VASA positive cells (e.g. arrowhead in panel 5a) did appear to have low levels of VASA in their cytoplasm and some nuclear OCT4. In the testes a the majority of germ cells (gonocytes, [8]) containing intense immunoexpression of OCT4 were located in the central portion of the seminiferous cords and these cells were VASA negative (Figure 5b). As in the ovary germ cells that contained intense cytoplasmic staining for VASA were OCT4 negative, these were often found in groups at the periphery of the cords. Nuclear expression of VASA was detected in some germ cells (Figure 5b, asterisks). As in the ovary rare germ cells containing very low levels of cytoplasmic VASA as well as low intensity staining for OCT4 were observed (Figure 5b arrowheads).

#### Differential and overlapping expression of DAZL and VASA

**Figure 6:**
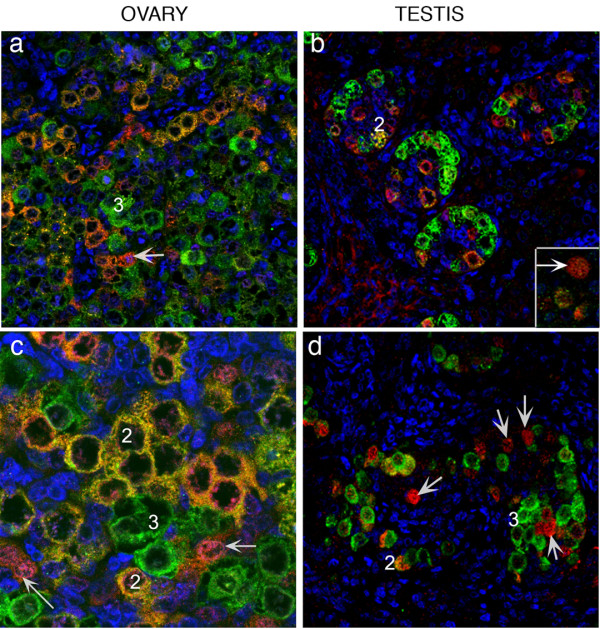
**Co-localisation of DAZL and VASA in 2nd trimester ovary and testes**. In the 2nd trimester (ovary, a, 17 wk; c, 14 wk; testes b, 15 wk; d, 16 wk) three populations of germ cells were identified based on their patterns of expression of DAZL (red) and VASA (green). 1stly a rare population of cells which had nuclear DAZL (arrows), 2ndly cells which co-expressed both proteins (population 2) and thirdly the most prevalent group which were VASA positive/DAZL negative (population 3). Although few cells in population 2 were detected in testes these germ cells were found as distinct groups within the ovary (see panel c).

The above data suggested that expression of DAZL and VASA was similar but not identical, in both ovary and testis. This was directly investigated by dual immunohistochemistry for DAZL and VASA in the 2nd trimester (Figure 6). This identified the presence of three populations of germ cells in both ovary and testis based on their patterns of expression of DAZL and VASA. Firstly a rare population of cells that expressed nuclear DAZL alone, secondly cells which co-expressed both proteins (population 2), and thirdly cells which were VASA positive/DAZL negative (population 3) which were the most prevalent. Although only few cells in population 2 were detected in testes these germ cells were found as distinct groups within the ovary (Figure 6b). Oocytes within primordial follicles expressed VASA with low/absent expression of DAZL.

**Figure 7:**
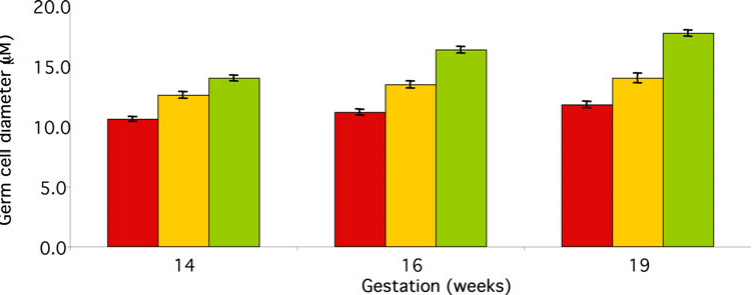
Ovarian germ cell diameter for cells expressing DAZL, VASA or both at 14, 16 and 19 weeks gestation. There were significant increases in diameter with gestation for each group (p = 0.001 for DAZL and VASA separately, p = 0.01 for cells expressing both). There were also significant differences (P < 0.01) between the 3 classifications of germ cell at each gestation. Mean ± sem, n = 20–120 per group.

In the ovarian samples the pattern of expression was further investigated by measuring germ cell diameter at 3 representative gestations (14, 16 and 19 weeks) as our previous studies have established that cell diameter provides an indication of the maturational status of the human fetal female germ cell [23]. This demonstrated that DAZL-predominant germ cells were smaller than germ cells showing marked expression of both proteins, which were in turn smaller than germ cells expressing VASA alone (Figure 7). These differences were significant at each gestational age examined (P < 0.001). This analysis also demonstrated that germ cells expressing only DAZL showed an increase in diameter from 10.6 ± 0.2 to 11.8 ± 0.3 μm (P < 0.01) between 14 and 19 weeks gestation, the cells found in groups that expressed both DAZL and VASA increased from 12.6 ± 0.3 to 14.0 ± 0.4 μm (P = 0.001) and those predominantly expressing VASA from 14.0 ± 0.3 to 17.8 ± 0.3 μm (P < 0.001).

## Discussion

Germ cells within the male and female gonads develop from an apparently homogeneous bi-potential population of PGC. In both male and female human fetal gonads germ cells initially proliferate but thereafter their fates diverge with female germ cells entering meiotic prophase as early as 11 weeks gestation (9 weeks post conception; [24]) whereas meiotic entry is does not occur until puberty in males. In rodents male germ cells removed from the testicular environment and placed *in vitro *can spontaneously enter meiosis emphasising the common developmental potential of the germ cells in the two sexes and the critical role played by the testicular environment in modifying germ cell fate [25,26].

In the current study we have used OCT4 immunohistochemistry to identify the subpopulation of putative 'germ stem' cells in human fetal testes and ovaries. Studies from our own laboratory [8] have already established that expression of OCT4 is restricted to the gonocyte population of human testicular germ cells whilst others [27] have reported that this protein is localised to oogonia in the ovary. Expression of *DAZL *and *VASA *was compared to *OCT4 *as studies in knockout mice have reported that both genes are essential for germ cell maturation in that species [14,19]. Analysis of ovarian extracts revealed a striking increase in the total amount of both DAZL and VASA mRNA and protein in the ovary as development progressed into the 2nd trimester. A similar trend in expression was noted in the testes but was less striking. Expression of OCT4 was maintained in both ovary and testis at all gestational ages examined consistent with persistence of a putative germ 'stem cell' population for many months in the human gonads although the extent to which these cells can acquire/maintain pluripotency *in vitro *remains unresolved (reviewed in [28]). In the 2nd trimester testis all seminiferous cords contained a mixture of OCT4 positive and OCT4 negative germ cells a situation which is different to that in rodents where the expression of OCT4 is down regulated synchronously in all germ cells within the cords before birth [29,30]. In the human fetal ovary expression of OCT4 protein was restricted to oogonia in the peripheral zone of the organ, in agreement with the observations of Stoop *et al.*[9]. In mice expression of *Oct4 *mRNA is extinguished in a rostro-caudal wave coincident with meiotic entry of germ cells [31].

Co-incident and overlapping patterns of expression of these germ cell specific proteins were revealed by fluorescent co-immunolocalisation on fixed specimens from a range of gestational ages. Previous studies have immunolocalised DAZL in a small number of samples recovered in the 2nd trimester [32-34] but we believe this is the first study to document expression in 1st trimester samples and to compare expression with that of OCT4 and VASA. In the 1st trimester, DAZL was detected in the nuclear compartment in OCT4 positive germ cells in both sexes. In a previous study we immunolocalised DAZL to the nuclei of male germ cells in a single sample at 17 weeks gestation and in the cytoplasm of female germ cells at 15 weeks [33]. Other studies have claimed that DAZL is present in both the cytoplasm and nucleus of male germ cells at 20–21 weeks [34]. We have extended these observations and demonstrated that DAZL is localised to the nuclear compartment in OCT4 positive cells (gonocytes and oogonia). In the ovaries localisation of DAZL to the cytoplasmic compartment appeared to be transient occurring as OCT4 expression but thereafter the amount of protein declined and was low in the largest ovarian germ cells, i.e. those that had formed into primordial follicles and those approaching that stage. This was evident when a morphometric analysis was performed showing that the germ cells immunopositive for DAZL alone were smaller than those containing both DAZL and VASA, which were in turn smaller than those only immunopositive for VASA.

Two studies on human populations have provided preliminary evidence that expression of *DAZL *is important for normal functioning of the human germ line. The first study reported a strong association between several common single nucleotide polymorphisms (SNPs) and age at menopause in a sample population of 324 women [35]. In a second study a patient with premature ovarian failure at age 34 was found to contain a homozygous mutation of *DAZL *(Arg to Gly at 115) in a region of the protein critical for RNA binding [35]. The situation in men is complicated by the presence of multiple copies of the closely related *DAZ *gene on the Y chromosome, deletions of which have been frequently documented as a cause of male infertility [36]. However a patient with a homozygous mutation in *DAZL *(Asn 10 Cys) was reported to be azoospermic [35].

The marked increase in expression of *VASA *between 9 and 14 weeks gestation suggested to us that this was coincident with the entry of female germ cells into meiosis. However increases in both protein and mRNA were also demonstrated in male germ cells suggesting that the onset of expression of *VASA *was associated the maturation of the gonocytes into prespermatogonia rather than meiotic entry per se. In the ovary VASA was detected in the cytoplasm of in slightly larger, more mature germ cells than DAZL, and was also present in oocytes within primordial follicles. Previous studies that have detected expression of VASA in cytoplasm of germ cells within the fetal ovary and testes at 17 weeks gestation [9,21,37]. Co-staining of sections with OCT4 and VASA has extended these findings by demonstrating that VASA protein is not expressed in gonocytes or oogonia in the 1^st ^trimester and provides a useful method for identifying the different populations of germ cells present within the 2^nd ^trimester gonads.

Examination of the 3' UTR of the *Mvh *mRNA has revealed a number of putative Dazl-binding sites that are conserved between human, rat and mouse [38]. Our data provide some additional support for a role for DAZL in initiating expression of *VASA *although further studies are required preferably using isolated human germ cells. DAZL has also been implicated in the regulation of other conserved germ-cell RNA-binding proteins including PUM2 [39], the human homolog of *Pumilio *that is required for maintenance of germ line stem cells in *Drosophila *and *Caenorhabditis elegans*.

## Conclusion

By documenting the differential and partially overlapping patterns of expression of OCT4, DAZL and VASA proteins in the human fetal ovary and testis we have gained new insight into parallels in the maturation of germ cells in these two organs. Our findings clearly demonstrate changes in overall germ cell maturation with increasing gestation, and indicate a switch from *OCT4 *expression in the less mature germ cells (gonocytes, peripheral oogonia) to *VASA *expression in the more mature germ cells (prespermatogonia, oogonia in nests, oocytes). In testicular samples germ cells at different stages of maturation co-exist within the same seminiferous cords. Both male and female germ cells showed a similar pattern of change with development, despite the onset of meiosis in the female but not in the male, suggesting that although increases in expression of *DAZL *and *VASA *in the ovary may be related to meiotic entry there are underlying developmental patterns common to both sexes.

## Methods

### Tissue recovery and processing

Human fetal gonads were obtained following termination of pregnancy during the first (61–64 days gestation, n = 11) and 2nd trimesters (14 to 19 weeks gestation, n = 29). Women gave written consent according to national guidelines [40] and the study was approved by the Lothian Ethics Committee. Termination of pregnancy was induced by treatment with mifepristone (200 mg, orally), followed by misoprostol (Pharmacia, Surrey UK; 200 mg every 3 h, per vaginam). None of the terminations was for reasons of fetal abnormality, and all fetuses used in this study appeared morphologically normal. Gestational age was determined by ultrasound examination before termination and was confirmed by subsequent direct measurement of foot length in 2nd trimester samples. The sex of the 1st trimester fetal gonads was determined by PCR for the *SRY *gene as detailed in [8]. Testes and ovaries were dissected carefully from the fetal abdominal cavity and either snap frozen and stored at -70°C prior to extraction of total protein or RNA, or fixed in Bouins fluid for 2–3 h, stored in 70% ethanol and processed into paraffin wax using standard methods.

### RNA Extraction and RT PCR

Total RNA was extracted from fetal gonads using the RNeasy Mini Kit (Qiagen, UK) for mid trimester gonads and the RNeasy Micro Kit for 1st trimester gonads (with on-column DNase digestion Qiagen, UK) The RNA was primed for reverse transcription with oligo(dT) primer (Applied Biosystems, UK) at 65°C for 10 min. The entire reaction was added to a total volume of 38 μl containing dNTP to 1 mmol/l, dithiothreitol (DTT) to 10 mmol/l, 1× Expand Reverse Transcriptase (RT) buffer and 120 IU RNasin ribonuclease inhibitor (Promega Ltd, UK). One half (19 μl) of this reaction was added to 1 μl water (RT negative reaction) to act as a negative control to confirm the effiency of the DNase treatment. Fifty IU of Expand Reverse Transcriptase (Roche Diagnostics Ltd) was added to the remaining 19 μl (RT positive reaction) and both reactions were incubated for 1 h at 42°C. Reactions were stored at -20°C until required.

### Real-time quantitative PCR

Quantitative real-time RT-PCR was performed using the Lightcycler (Roche Diagnostics Ltd) as described previously [41]. Reverse-transcribed RNA samples were diluted in water as indicated below. One microlitre of diluted first-strand cDNA was added to a final volume of 10 μl containing 50 μg/ml non-acetylated bovine serum albumin and 0.5 μmol/l each of forward and reverse primer in 1× Platinum^®^SYBR Green qPCR SuperMix-UDG (Invitrogen. Paisley, UK). Amplification was continued for 45 cycles with signal acquisition at 84°C after each round of extension. Following amplification, continuous melt curve analysis was performed to ensure product accuracy and samples were analysed by agarose gel electrophoresis (data not shown) to confirm product size. Primer sequences are given in Table 1, Standard curves for *GAPD*, *OCT4*, *DAZL *and *VASA *were derived by making a series of dilutions (1 in 5 to 1 in 10000) of first-strand cDNA from a mid trimester ovary. The number of cycles needed to yield a fluorescent signal above background (the cross-over point, Cp) at each dilution was plotted against the log of relative concentration using LightCycler Software (Molecular Dynamics Ltd, UK). The dilutions yielded a straight line for each product, confirming that Cp is a good indicator of target concentration across at least 2 orders of magnitude. The slopes of these curves are a measure of the efficiency of the PCR, which gave an amplification rate of 1.8-fold/cycle for *GAPD *and *DAZL*, and 1.7-fold/cycle for *OCT4 *and *VASA*. All gene amplification reactions were performed in triplicate. Calculations of mRNA concentration were made relative to *GAPD*.

**Table 1:** Primers used for quantitative PCR

Gene	Sequence	Accession No.	Product Size
h*GAPD*F	GACATCAAGAAGGTGGTGAAGC		
h*GAPD*R	GTCCACCACCCTGTTGCTGTAG	NM_002046	212 bp
h*DDX4MVH *F	AAGAGAGGCGGCTATCGAGATGGA		
h*DDX4MVH *R	CGTTCACTTCCACTGCCACTTCTG	NM_024415	238 bp
h*OCT4*F	ACATCAAAGCTCTGCAGAAAGAAC		
h*OCT4*R	CTGAATACCTTCCCAAATAGAACCC	NM_002701	126 bp
h*DAZL*F	GAAGGCAAAATCATGCCAAACAC		
h*DAZL*R	CTTCTGCACATCCACGTCATTA	NM_ 001351	186 bp

### Western blot analysis

Fetal gonads were homogenised in 1× RIPA buffer containing: 25 mM Tris, 1% Triton, 0.05% sodium deoxycholate, 0.1% SDS and 150 mM NaCl. Total protein was measured using the Protein Assay DC Kit from Bio-Rad (Hemel Hempstead, Herts, UK). Samples were denatured in 1× reduced sample buffer containing: 625 mM Tris (pH 6.8), 5% glycerol, 2% SDS, 0.0025% bromophenol Blue, 2.5% β-mercaptoethanol and 5 μg of total protein was loaded onto individual wells in a SDS-PAGE gel made with 10% (w/v) acrylamide; samples of pre-stained protein size markers were run on each gel (SeeBlue plus 2 prestained standard, Invitrogen, UK). Following separation of proteins they were transferred onto PVDF Immobilon-Fl membranes (Millipore UK Ltd, Watford, Herts); non-specific binding sites were blocked by incubating membranes in Odyssey blocking buffer (LI-COR, Lincoln, Nebraska, USA). All membranes were incubated with primary antibodies diluted in Odyssey blocking buffer overnight at 4°C. Proteins were detected using rabbit-anti-VASA (DDX4MVH, Abcam, Cambridge UK) diluted 1 in 500 together with mouse anti-β tubulin (cat no. T-4026, Sigma) diluted 1 in 300, mouse anti-DAZL (gift from Professor Howard Cook, MRC Human Genetics Unit, Edinburgh) diluted 1 in 500, with rabbit anti-β-tubulin (cat no. sc-9104, Santa Cruz) diluted 1 in 1000, or with goat anti-OCT4 (Santa Cruz) together with rabbit anti-β-tubulin (Santa Cruz) diluted 1 in 1000. Membranes were washed in PBS containing 0.1% Tween and bound antibodies were detected using fluorescently labelled secondary antibodies: goat anti-rabbit 680 (Invitrogen, Paisley, UK) and goat anti-mouse 800 (Invitrogen, Paisley, UK) for VASA and β-tubulin respectively; goat anti-mouse 680 (Invitrogen, Paisley, UK) and goat anti-rabbit 800 (Rockland, USA) for DAZL plus β-tubulin; while OCT4 and β-tubulin, were detected using donkey anti-goat 680 (Invitrogen Paisley, UK) and goat anti-rabbit 800 (Rockland, USA). Secondary antibodies were all diluted 1 in 10000 in Odyssey blocking buffer and incubated for 1 h at room temperature; bound fluorescent secondary antibodies were visualised using a LI-COR-Odyssey Infrared Imager (LI-COR Biosciences, Lincoln Nebraska).

### Immunofluorescence co-localisation

Immunolocalisation was carried out using standard methods [8]. Briefly, 5 μm sections were mounted onto electrostatically charged microscope slides (VWR, Poole, UK), dried overnight, then dewaxed and rehydrated using conventional methods. Heat-induced antigen retrieval was performed by placing slides in a pressure cooker in 0.01 M citrate pH 6 for 5 min on full power with 20 min standing prior to cooling. Slides were transferred into Tris-buffered saline (TBS; 0.05 M Tris, 0.85% NaCl (pH 7.6)) for 5 min and blocked in TBS with 20% normal serum (Diagnostics Scotland, Carluke, UK), 5% BSA and avidin (0.01 M, 15 min) then biotin (0.001 M, 15 min; both from Vector Laboratories, Peterborough, UK) with washes in TBS in between. Primary antibodies and detection are listed in Table 2. All primary antibodies were incubated on sections overnight at 4°C. Biotinylated secondary antibodies were incubated for 30 min at room temperature. Peroxidase-labelled and direct conjugate antibodies were incubated for 1 h at room temperature. Tyramide was left for 10 min and all slides were counterstained with DAPI (Sigma, cat no D9542) diluted 1/2000 in PBS. Mounted slides were visualised using a laser scanning confocal microscope (Zeiss).

**Table 2:** Antibodies used for immunofluorescent immunohistochemistry

**Antibody**	**Dilution**	**Blocking Serum**	**Detection**
DAZL+VASA	DAZL 1/500+VASA 1/300	goat	GAMB+Streptavidin-546 (DAZL) GAR-488 (VASA)
VASA + OCT 4	VASA 1/300+OCT 41/200	chicken	CARB+Streptavidin-488 (VASA) CAGP + Tyr-Cy3(OCT4)
DAZL + OCT 4	DAZL 1/500 + OCT 4 1/200	rabbit	RAMB+Streptavidin 546 (DAZL) RAGP+TyrCy3 (OCT4)

VASA, ab13840, Abcam, Cambride UK

OCT4, Sc8629, Santa Cruz, CA, USA

DAZL MCA 2336, Serotec, Oxford, UK

GAMB = goat anti-mouse biotinylated secondary antibody (DAKO, UK) diluted 1/500

Streptavidin-546 (Invitrogen, UK) diluted 1/200

GAR-488 = goat anti-rabbit-488 (Invitrogen, UK) diluted1/200

CARB = chicken anti-rabbit biotinylated (Santa Cruz, CA, USA) diluted 1/200

RAMB = rabbit anti-mouse biotinylated (DAKO, UK) diluted 1/500

CAGP = chicken anti-goat peroxidase (Santa Cruz, CA, USA) diluted 1/200

Tyr-Cy3 = Tyramide Cy3 (Molecular Probes, UK) diluted 1/5.

### Statistical analysis

Three representative non-adjacent tissue sections from ovaries at gestational ages 14, 16 and 19 weeks immunostained for DAZL and VASA were analysed. The cell diameter of all immunopositive germ cells on each section was calculated as the average of two orthogonal measurements and classified as expressing DAZL, VASA or both. Data were analysed by analysis of variance with Tukey-Kramer post hoc testing.

## Authors' contributions

RAA and PTKS designed the study and wrote up the manuscript. GC and NF performed QRTPCR, Western and immunohistochemical analysis and acquired images on the confocal microscope. SC performed tissue collections. All authors read and approved the final manuscript.
